# Interventions in the first 1000 days to prevent childhood obesity: a systematic review and quantitative content analysis

**DOI:** 10.1186/s12889-022-14701-9

**Published:** 2022-12-16

**Authors:** Celia Pérez-Muñoz, Jesús Carretero-Bravo, Esther Ortega-Martín, Begoña Ramos-Fiol, Bernardo Ferriz-Mas, Mercedes Díaz-Rodríguez

**Affiliations:** 1 Facultad de Enfermería Y Fisioterapia, University of Cádiz, Ana de Viya 52, 11009 Cádiz, Spain; 2 Andalusian Health System, Cádiz, Spain

**Keywords:** Childhood obesity, First 1000 days, Intervention

## Abstract

**Background:**

Childhood obesity poses a global health challenge. In recent years, there has been an increase in interventions that begin in pregnancy, putting the concept of early programming and early risk factors into practice. The present study aims to update the findings regarding interventions in the first 1000 days of life.

**Methods:**

A systematic review based on the PRISMA guidelines was carried out in PubMed, WoS, Scopus and CINAHL to obtain the articles to be analysed. We included those studies published between 2016 and 2021. Human interventions that started within the first 1000 days of life and acted on at least one programming factor were included. Once selected, coding and quantitative content analysis was carried out to obtain a profile of the interventions during the first 1000 days.

**Results:**

From all screened articles, 51 unique interventions, which met the selection criteria, were included. The majority of interventions (81%) took place in high-income areas. Almost all (86%) were targeted at the general population. The majority (54%) started in the second trimester of pregnancy. A clear majority (61%) ended at the time of birth. 44% of the interventions included all pregnant women. Only 48% of these interventions were focused on improving the nutritional status of the offspring in the short term. Most interventions collected the baby's weight at birth (68%).

**Conclusions:**

It can be concluded that current interventions are not covering as many aspects as they should. Future research should be conducted more frequently in developing countries and target disadvantaged groups. These interventions should include all pregnant women, regardless of their nutritional status, aiming to cover as many programming factors as possible and extending through the first 1000 days of life, with body mass index or skinfolds as measures of effectiveness during this period.

**Supplementary Information:**

The online version contains supplementary material available at 10.1186/s12889-022-14701-9.

## Introduction

Childhood obesity poses a global health, policy and research challenge [1]. In 1975, 32 million children under five years of age worldwide had overweight or obesity, growing to 42 million by 2020 [1, 2]. Even more worryingly, obesity affects children as young as two years old [1].

Besides being a problem by itself, in the short and medium-term, childhood obesity is associated with multiple diseases normally developed in adults, such as type 2 diabetes mellitus, hypertension, non-alcoholic fatty liver disease and dyslipidaemia [3, 4]. In the long term, children suffering from obesity have an increased risk of developing type 2 diabetes, hypertension, dyslipidaemia and atherosclerosis in adulthood [3]. According to a systematic review, most children with obesity become adults with obesity [5]. Beyond physical illnesses, excess weight in childhood has consequences on the mental health of children who suffer from it, as well as on their academic performance [3]. Among the most frequent psychosocial problems we find low self-esteem, anxiety, depression and a lower quality of life [3].

This fact, coupled with associated comorbidities, is why the increasing prevalence of obesity in childhood poses a challenge to health systems, among others [6]. Although it may seem a problem mainly associated with high-income countries, according to WHO estimates, of the 42 million children under five years of age who have excess weight globally, 35 million belong to developing countries [1]. On the other hand, studies reveal inequalities between different social classes, with children from the most disadvantaged social groups being more affected by having excess weight [7, 8].

As early as the first half of the twentieth century, it became known that early exposure to environmental factors could have a long-term effect on the health of offspring [9, 10]. Subsequently, it was thanks to the formulation of the hypothesis of the foetal origin of adult diseases that a direct link was established between the incidence of certain adult diseases and an environment of malnutrition during pregnancy [11].

Despite efforts to reverse this situation, the prevalence remains high, suggesting that the current approach to interventions is inadequate. The concept of early programming has taken centre stage in recommendations for preventing childhood obesity [12, 13]. Early programming is defined as the process by which one exposure to a series of external factors, in early stages of the development, produces permanent changes in the body that can influence future health [14]. Research into the effect of a range of environmental factors in the first 1,000 days of life, including pregnancy and the first two years of life, on developing non-communicable diseases has led to changing strategies [15]. In recent years, there has been an increase in interventions covering risk factors, that begin during pregnancy, putting the concept of early programming into practice [16] and are leading the way in preventing childhood obesity.

Many risk factors at this stage of development have been shown to affect future childhood obesity: maternal weight at the start of pregnancy, gestational weight gain (GWG), gestational diabetes mellitus (GDM), smoking, maternal undernutrition, caesarean section, high protein intake, no or short breastfeeding, vitamin D deficiency, excessive weight gain during the first year of life, and others such as sleep quality, screen viewing, attachment, appetite control, physical activity or complementary feeding [17–21]. In addition, it is common for the same child to have more than one of these factors, which has been shown to have a cumulative effect, increasing the risk of obesity [22, 23]. However, current interventions generally do not cover all of these factors but focus on some of them, especially through nutrition and physical activity [24], leaving aside others as necessary as sleep or the use of screens, among others. With this in mind, it is essential to analyse the type of interventions that are being implemented in the first 1000 days of life to prevent childhood obesity, what risk factors are covered, where they are delivered, how the intervention is delivered and the type of mothers who are recipients of the intervention. Previous reviews up to 2016 have done a similar analysis of these interventions [25–28]. However, these reviews cover interventions targeting a broader age range, including birth to 18 years. The present study aims to update the findings regarding interventions in the first 1000 days of life by reviewing the scientific literature in the main databases and conducting a quantitative analysis of the content of these databases [28–31]. With this, we will be able to outline the main characteristics of the interventions and identify gaps and shortcomings to target future interventions to address these possible deficiencies.

## Methodology

A systematic review of the literature based on the guidelines for Systematic Reviews and Meta-Analyses (PRISMA) was carried out to obtain the articles to be analysed [32]. Articles were screened, selected against the inclusion criteria, and selected those finally included for analysis. The protocol had previously been defined, detailing the different phases of the process and the inclusion and exclusion criteria. The protocol was registered in the PROSPERO platform on 02 November 2021 (CRD42021282951).

We included those studies published between 01 January 2016 [25, 29] and 31 December 2021. Studies published in English and Spanish were included, languages the authors were fluent in and did not require external translation. Human interventions that started within the first 1000 days of life and acted on at least one programming factor were included [17–21]. Articles were excluded if they were not interventions, were not original articles or were only the publication of a protocol, with no publications being carried out at the review date. Following Cochrane guidelines for systematic reviews, only one article per study was included [33]. If more than one article per study was found, the one with the most recent data on offspring follow-up was included to avoid double information when analyzing the content. Even so, when it has been necessary to consult the other articles to obtain complete information, it has been done.

Once selected, coding and quantitative content analysis was carried out to obtain a profile of the interventions during the first 1000 days.

### Search strategy and initial screening

The search was carried out by two researchers in four databases: PubMed, WoS, Scopus and CINAHL. These were the databases selected as they are the most commonly used in the most recent systematic reviews. For this purpose, the conjunction of different terms was adapted to each database to obtain the best results. The complete search strategy can be seen in Additional file 1. The terms used were derived from the research question and were related to obesity, the first 1000 days and interventions. The filters offered in the databases regarding publication date, language and human research were used.

The searches were conducted during September 2021, and a final search was conducted in January 2022 to cover articles published between September and December 2021. This last search found only 1,903 new articles, of which, after the screening, none were included in the final analysis as they did not meet the inclusion criteria.

**Fig. 1:**
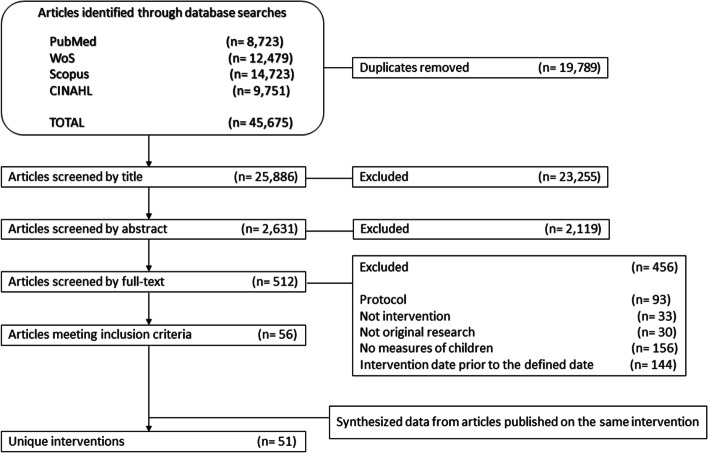
PRISMA flow diagram for identifying and screening eligible childhood obesity prevention interventions

Figure 1 summarises the process from search to final inclusion of the selected articles. A total of 45,675 articles were identified, of which 19,789 were eliminated as duplicates using a reference manager. The remaining 25,886 articles were screened by title, eliminating all those not written in the target language, did not involve humans, had other objectives or were not original research articles. Finally, 2,631 studies were selected in this phase to continue the process.

### Application of eligibility criteria

The articles selected so far were reviewed by abstracts. Eligibility criteria were applied, which were guidelines for screening based on study abstracts. Interventions were included if they had their start in the first 1000 days of life, collected some offspring variable and aimed, directly or indirectly, to prevent childhood obesity in the short or long term.

In this step, 2,119 articles were eliminated, resulting in 512 studies being selected for the next stage. In the full-text reading, the same eligibility criteria were applied, checking for any remaining doubts by simply reading the abstract and confirming that all inclusion criteria were met in detail. Finally, 456 articles were excluded at this stage, so 56 studies were included in the review. The reasons for exclusion at this stage were: being only the protocol of intervention, not being an intervention, not being original research articles, not collecting any offspring variables, or being an intervention before the defined date. The latter case refers to interventions conducted well before the stated publication date and whose results were published and included in previous reviews. The reviewers reached a 92% level of agreement, while discrepancies were resolved by discussion.

The 56 selected articles were reviewed according to the first author's surname, name of the intervention and country where it was developed to identify duplicate studies from the publication of several articles on the same intervention. Finally, five articles were eliminated, resulting in a sample of 51 articles belonging to 51 unique interventions included for analysis. The complete list of included articles can be found in Additional file 2.

### Data extraction

We used conventional content analysis methodology. We extracted and analyzed article, intervention and participant characteristics. We developed a codebook to standardize the coding process [28–31]. This codebook was tested by authors. Three articles, identified in the initial search and excluded during the screening, were used to test the coding that had been established. Finally, four articles included in the final sample (approximately 10%) were used to confirm the codes.

#### Inter-rater reliability

Two observers were responsible for data extraction. To test inter-rater reliability, 26 articles were randomly selected from those that would ultimately form part of the review (50%), and inter-rater agreement was tested using Cohen's kappa coefficient [34]. Two rounds of coding training were conducted to achieve sufficient reliability. Reliability was assessed for each variable. The mean kappa score for all variables was 0.80, while the percentage of simple agreement reached 94.4%. Variables with a minimum kappa of 0.7 were considered good reliability [35]. Two variables had a kappa value below this score: What does it aim to address? (0.66) and Variables Collected Baby (0.61), but as they had a high total agreement, and were variables with more than four categories they were considered for analysis, as the kappa coefficient is difficult to interpret as the number of categories increases [35, 36].

Then, the reviewers carried out the coding of a part of the final sample of 51 articles, each one separately.

#### General characteristics of the interventions

The general characteristics of the articles and the interventions they showed were coded. For the characteristics of the articles themselves, the year of publication and the main subject of the journal in which they were published were coded. Six categories were established for the year of publication (2016, 2017, 2018, 2019, 2020 and 2021), in the same way as for the main topic of the journal of publication (Nutrition, Obesity, Childhood obesity, Gynaecology and obstetrics, Medicine and Paediatrics).

Concerning the general characteristics of the interventions, we coded the geographical region in which it took place, the type of population targeted, the setting in which it took place and how it was developed.

Six categories were defined in geographic region, including Europe/UK, USA, Australia/New Zealand, South America, Asia and Africa, thus covering all possible geographic areas. The target population was coded into general, ethnic/racial, and low-income groups.

Because the interventions being evaluated in this article started in the first 1000 days of life, the setting in which they have developed was coded into four categories (community-based, health care, home-based, multi-setting) since it is not possible to develop them in other settings such as schools. Finally, all interventions were concentrated in two established categories (community-based and health care).

The mode in which the intervention took place was also coded. Three categories were established: in person, with technology, and both.

#### Specific characteristics of the interventions

All characteristics of the interventions of interest for profiling and gap identification were coded to guide future research. In this way, the time at which the intervention begins was coded into three categories: first trimester, second trimester and third trimester, given that all the interventions started during the period of pregnancy. As for the end of the intervention, six categories were established according to whether the interventions ended during pregnancy, at birth, during the first year, at the first year, at the second year or after the second year.

Five categories were coded for the mothers included in terms of their body mass index (BMI) at the start of pregnancy: all pregnant women, pregnant women who are overweight, pregnant women who are obese, only pregnant women who are normal-weight, or pregnant women with a clinical history of GWG, GDM or macrosomic offspring. About the aspect that the intervention addresses intending to improve it, four categories were coded: excessive weight gain, gestational diabetes mellitus, pregnancy outcomes in general and nutritional status of the offspring. On the other hand, how the intervention addresses these aspects was categorised, resulting in six categories: nutrition, physical activity, nutrition and physical activity, general lifestyle including breastfeeding promotion, general lifestyle including breastfeeding and sleep, and supplement use.

Five categories were coded for the main variables collected: GWG, DMG, measures of children, breastfeeding and pregnancy outcomes. The measures collected in the offspring were classified into birth weight, and BMI at different times or skinfolds. Finally, the time these measures were taken was coded into four categories: at birth, during the first year, from birth to two years and after two years.

### Data synthesis and analysis

First, the data was cleaned. In cases where data were missing or in doubt, the entire article was reviewed to confirm the information. The unit of analysis is the intervention, so a denominator of 51 interventions was used to assess the characteristics of the interventions. The tables presented show the number of interventions, accompanied by the percentage of the sample in brackets, that meet a given characteristic. Inter-rater reliability was performed with SPSS (version 24).

## Results

From all screened articles, 51 unique interventions, which met the selection criteria, were included. The general characteristics of these interventions are summarised in Table 1.

**Table 1:** General characteristics of childhood obesity prevention interventions published from 2016 to 2021 (*n* = 51)

	n (%)
Publication Year	
2016	12 (24)
2017	5 (10)
2018	8 (16)
2019	9 (18)
2020	7 (13)
2021	10 (19)
Topic of the Journal of publication	
Nutrition	14 (28)
Obesity	5 (10)
Childhood obesity	4 (8)
Gynaecology	13 (25)
Medicine	10 (19)
Paediatrics	5 (10)
Geographic region	
Europe/United Kingdom	12 (24)
United States	17 (33)
Australia/New Zealand	12 (24)
South America	3 (5)
Asia	6 (12)
Africa	1 (2)
Targeted population	
General population	44 (86)
Racial/ethnic groups	6 (12)
Low-income groups	1 (2)
Setting	
Community-based	16 (31)
Health care	35 (69)
Mode of delivery	
In-person	31 (61)
Using technology	2 (4)
Both	18 (35)

As for the year of publication, we observed a peak of interventions published in 2016 (24%) that declined in the following years, although it increased again in the last years, 2019 and 2021. We refer to the main topic of the journals in which the selected interventions are published. In that case, we found that most interventions were published in gynaecology (25%) and nutrition journals (28%), with only 8%published in specific journals of childhood obesity or 10% in obesity journals.

Focusing on the geographical region in which the intervention took place, a majority of interventions (81%) took place in high-income areas, such as the USA (33%), Australia/New Zealand (24%) and Europe/UK (24%). There were very few interventions in developing areas such as Africa (2%), South America (5%) or Asia (12%) and nothing in Central America.

In terms of the target population of these interventions, almost all (86%) were targeted at the general population, with very few interventions explicitly targeting ethnic or racial groups (12%) or the lower-income population (2%). On the one hand, interventions targeting racial or ethnic groups were developed only in the USA, accounting for 33% of all interventions developed in this country. On the other hand, the only intervention aimed at the lower-income population had been developed in South America.

Regarding the setting of the interventions included in this review, we found that most interventions (69%) had been developed in health care settings.

Finally, most interventions continued to be carried out face-to-face (61%). However, there was a growing tendency to use technology in combination with face-to-face development of interventions (35%).

The characteristics of the interventions are summarised in Table 2.

**Table 2:** Intervention characteristics of childhood obesity prevention interventions published from 2016 to 2021 (*n* = 51)

	n (%)
Start of the intervention	
First trimester	14 (28)
Second trimester	28 (54)
Third trimester	4 (8)
Postpartum	5 (10)
End of the intervention	
During pregnancy	5 (10)
At birth	31 (61)
During the first year	6 (12)
At first year	4 (8)
At second year	2 (4)
> 2 years	3 (5)
Included mothers	
All pregnant	22 (44)
Normal weight	5 (10)
With obesity and overweight	15 (29)
With obesity	6 (12)
With clinic antecedents	3 (5)
Targeted issue	
Gestational weight gain	13 (25)
Gestational Diabetes Mellitus	3 (5)
Pregnancy outcomes	11 (22)
Children nutritional status	24 (48)
Targeted behavior^a^	
Nutrition	8 (16)
Physical Activity	11 (22)
Nutrition and Physical Activity	17 (33)
Lifestyle (including breastfeeding)	13 (25)
Lifestyle (including sleep)	3 (5)
Use of supplements	2 (4)
Main outcomes	
GWG	18 (36)
GDM	10 (19)
Breastfeeding	6 (12)
Measures of children	14 (28)
Pregnancy outcomes	3 (5)
Measures of children	
Birthweight	35 (68)
Body mass index (BMI)	8 (16)
Skinfolds	8 (16)
Moment of children measures	
Birth	30 (59)
During the first year	10 (19)
Birth to 2 years	4 (8)
> 2 years	7 (14)

^a^Groups are not mutually exclusive thus, totals may exceed 100%

In terms of the time these interventions began, the majority (54%) started in the second trimester of pregnancy, i.e. from the 14th week. Only 28% of the interventions began before this time, during the first trimester.

There were more differences in the timing of the end of the interventions. A clear majority (61%) ended at the time of birth. Of the remainder, 10% ended before pregnancy came to an end, while 12% ended at different times during the first year of life. 8% of interventions continued into the second year of life and ended at some point during the second year of life, while only 4% ended at the age of two years. On the other hand, only 5% of the interventions continued after the second year of life.

Regarding the group of women targeted by the intervention, in terms of BMI at the start of pregnancy, 44% of the interventions included all pregnant women. In comparison, 29% included only those with excess weight, i.e. with overweight or obesity. 12% of the interventions targeted only women with obesity, and 10% targeted only women with average weight. Finally, 5% of the interventions targeted mothers with a medical history of gestational diabetes, a newborn with macrosomia or some other pregnancy complication.

We found different categories when analysing the issue on which the interventions were focused. Only 48% of these interventions were focused on improving the nutritional status of the offspring in the short term. 25% were focused on controlling the weight gain of pregnant women during this period in order to avoid excessive weight gain. 22% of interventions aimed to improve pregnancy outcomes, while 5% were focused on preventing the development of gestational diabetes mellitus.

Regarding the targeted behavior, most interventions were focused on nutrition and physical activity at the same time (33%). While 25% addressed lifestyle to improve health, including breastfeeding, only 5% also included sleep as a determining factor.

In terms of the main variables collected, there were many interventions whose main outcome measure was excessive weight gain (36%). The rest focused on collecting the development of gestational diabetes mellitus (19%), initiation of breastfeeding (12%), anthropometric measures in offspring (28%) or pregnancy outcomes in general (5%).

Most interventions collected the baby's weight at birth (68%). Only 16% took BMI measurements, which was valid for skinfolds. As for the timing of these measurements, 19% took them at different times during the first year of life, only 8% of the interventions took them at two years of age, while 14% took them after this age.

Table 3 represents a cross-tabulation of the mothers including the targeted behavior (referred to the parents or child, depending on the intervention), the main variables, the variables collected from the baby and the time of collection of these variables according to the aspect addressed.

**Table 3:** Included mothers, targeted behavior, main outcomes, children’s measures and moment of collection depending on the targeted issue (*n* = 51)

	Targeted issue		
n (%)	GWG + GDM	Pregnancy outcomes	Children nutritional status
Included mothers			
All pregnant	8 (50)	5 (45)	10 (42)
Normal weight	1 (6)	3 (28)	0 (0)
With obesity and overweight	5 (32)	1 (9)	9 (38)
With obesity	1 (6)	2 (18)	3 (12)
With clinic antecedents	1 (6)	0 (0)	2 (8)
Targeted behavior			
Nutrition	3 (19)	0 (0)	5 (21)
Physical Activity	5 (32)	3 (28)	3 (12)
Nutrition and Physical Activity	7 (43)	6 (54)	4 (17)
Lifestyle (including breastfeeding/sleep)	1 (6)	2 (18)	10 (42)
Use of supplements	0 (0)	0 (0)	2 (8)
Main outcomes^a^			
GWG	16 (100)	5 (45)	4 (17)
GDM	9 (56)	7 (63)	0 (0)
Breastfeeding	0 (0)	2 (18)	4 (17)
Measures of children	0 (0)	5 (46)	13 (54)
Pregnancy outcomes	0 (0)	3 (28)	3 (12)
Measures of children			
Birthweight	14 (88)	6 (55)	8 (33)
Body mass index (BMI)	2 (12)	1 (9)	11 (46)
Skinfolds	0 (0)	4 (36)	5 (21)
Moment of children measures			
Birth	14 (88)	7 (63)	9 (38)
During the first year	1 (6)	3 (28)	6 (25)
Birth to 2 years	0 (0)	1 (9)	3 (12)
> 2 years	1 (6)	0 (0)	6 (25)

^a^Groups are not mutually exclusive thus, totals may exceed 100%

It was observed that those interventions that focused on controlling GWG or GDM, included mostly (50%) all types of women in terms of BMI. However, a high percentage (32%) of interventions included only those women with excess weight. They were mainly interventions that combined nutrition and physical activity as tools (43%). Finally, they were interventions that primarily collected the GWG of mothers (100%) and the birth weight of offspring (88%).

Interventions that aimed to improve pregnancy outcomes generally were more diverse in terms of the BMI of the mothers included, although the majority (45%) included all types of women. 54% of these interventions addressed nutrition and physical activity together. As for the collection of variables, there was diversity among the interventions, finding those that collect GWG, GDM, and different measures in the offspring or at the end of pregnancy.

Finally, interventions focused on improving the nutritional status of offspring mainly included all mothers (42%) but remains high the number of interventions that only included those mothers who have obesity or overweight (38%). They improved their overall lifestyle, including breastfeeding or sleep, to achieve their goals (42%). Most of these interventions collected measures of offspring at birth (38%). These measures included skinfolds and BMI, as well as birth weight.

**Fig. 2:**
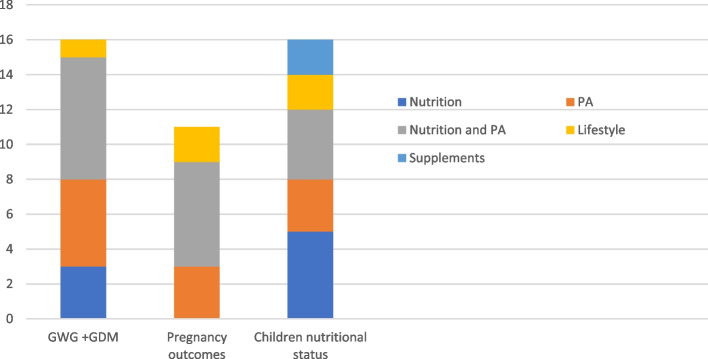
Distribution of the targeted behavior according to the targeted issue of the interventions developed

Figure 2 shows the type of developed interventions, depending on the targeted issue. It highlights how most interventions focused on nutrition or physical activity while only those that focused on improving the nutritional status of offspring covered lifestyle in general.

## Discussion

Following the systematic review process, this study shows the results of a quantitative content analysis of interventions to prevent childhood obesity, directly or indirectly, published between 2016 and 2021 to obtain a profile and identify gaps to guide future research.

Gaps have been identified concerning the geographical region where the interventions were developed and the social groups targeted. On the other hand, gaps have been identified in terms of the start and end time of the interventions, the BMI of the mothers included, the targeted behavior and the factors it addressed, and the measures collected in the offspring.

In terms of geographical region, it has been found that most interventions in recent years were developed in North America, Oceania and Europe. Previous studies highlighted the need for such interventions in developing countries where the prevalence of childhood obesity remains very high [1, 37].

Similarly, it has been noted that most interventions were targeted at the general population, which is necessary given the data on childhood obesity worldwide. However, there is a recognized need to develop interventions targeted at the most disadvantaged groups, be they racial/ethnic or economically disadvantaged groups, as this is where inequalities originate, increasing the prevalence of childhood obesity [38–42].

When it comes to the timing of interventions, the answer is clear: the earlier, the better [43]. However, it has been observed that most interventions started in the second trimester of pregnancy, some even after birth. Interventions should begin from the first trimester, spanning the entire pregnancy, a period linked to future obesity in offspring [44, 45]. Even so, we know that it is not easy since in the first trimester, mothers are still learning about their pregnancy and adapting to the symptoms. Still, efforts should be directed towards early initiation of interventions when possible. Similarly, the time of termination of interventions has been positioned, in most of the interventions analysed, at the time of birth, with few interventions continuing into the postnatal period and only five, of the 51 included, ending at two years of age or more, covering the period of the first 1000 days of life. It has been shown that this is the period with best plasticity and where the actions carried out will have more significant effects on the future since once this period is over, plasticity decreases so that the actions carried out will not be as successful [44]. Moreover, the cumulative effect of many exposures over this period results in poor future health [13]. Therefore, the entire period of the first 1000 days should be covered to minimise exposures. As it is a long period of time, adherence is difficult. However, this is an age when parents are involved in their children's health, which facilitates adherence.

On the other hand, interventions should be targeted at all pregnant women, regardless of their weight at the start of pregnancy, as weight gain during this period is an early programming factor affecting offspring [46–49]. This review found that many of the interventions included all pregnant women (44%), but there were still many interventions that focus only on those who are overweight (29%) or only on those who are obese (12%) at the start of pregnancy. Women who have obesity and overweight should have a particular focus since their offspring are at increased risk [46–49]. However, to cover as many factors as possible interventions should target all women.

In terms of the factors covered by current interventions, it has been found that almost all interventions targeted a single factor (GWG, GDM) or generally improved the pregnancy outcomes or nutritional status of future offspring. However, only nutrition, physical activity or a combination of both are covered, while very few interventions included factors such as breastfeeding promotion or sleep hygiene. Interventions should be comprehensive, targeting all possible programming factors, as there is often more than one factor in the same individual, which has been shown to have a cumulative effect on future obesity risk [43, 50, 51].

Finally, concerning measures collected in offspring, as a measure of the effectiveness of interventions, most of the studies analysed collected weight at birth, which is the end of the intervention. As mentioned above, interventions should be continued until two years, so the most effective measures would be BMI or skinfolds at this age [52]. BMI is the most widely used. Although it has some limitations, skinfold measurement could overcome these limitations by allowing the estimation of body fat percentage [52].

Previous systematic reviews of interventions in the first 1000 days of life [26, 27] found efficacy for interventions that began during pregnancy and continued postnatally. Of these, those that only intervened in one aspect were effective. Those based on home visits and group sessions focusing on diet, infant feeding practices and physical activity were effective. On the other hand, reviews focusing on the content analysis [24, 28] found that most interventions were developed in the general population without taking into account ethnic or racial minorities and concentrated on a single factor, mainly diet and physical activity just as we have done.

## Limitations

The present study has some limitations that should be taken into account. Firstly, the quality of the included studies was not assessed, as the aim of the review was not to evaluate their effectiveness but to profile the interventions. Secondly, we did not include studies that only presented an intervention protocol or without any related publication with outcomes, to avoid biasing the profile obtained with protocols that were not carried out. This fact does not allow interventions already being carried out appear in this review and it should appear in future reviews.

## Conclusions

From the analyses presented here, it can be concluded that current interventions are not covering as many aspects as they should. Future research should be conducted more frequently in developing countries and target disadvantaged groups. In addition, these interventions should include all pregnant women, regardless of their nutritional status, aiming to cover as many programming factors as possible and extending through the first 1000 days of life, with BMI or skinfolds as measures of effectiveness during this period. Ensuring that the interventions that are carried out cover all the aspects mentioned above will lead to better prevention of childhood obesity and an improvement in the epidemic that adult obesity entails.

## Supplementary Information

**Additional file 1.** Search strategy for databases to identify eligible interventions during the first thousand days to prevent childhood obesity published between 2016 and 2021.

**Additional file 2.** List of eligible articles published between 2016 and 2021 detailing an intervention during the first thousand days to prevent childhood obesity.

## Data Availability

The data extracted in the current study is available from the corresponding author on reasonable request.

## Abbreviations

US: United States
WHO: World Health Organisation
GWG: Gestational weight wain
GDM: Gestational Diabetes mellitus
PRISMA: Preferred Reporting Items for Systematic Reviews and Meta-Analysis
UK: United Kingdom
BMI: Body mass index
PA: Physical Activity
