# Supplementary material for: Interventions in the first 1000 days to prevent childhood obesity: a systematic review and quantitative content analysis
Source: BMC Public Health. 2022 Dec 16;22:2367. doi: 10.1186/s12889-022-14701-9 (PMC9758903; doi:10.1186/s12889-022-14701-9)
Supplement: Supplementary file 1 — Additional file 1. Search strategy for databases to identify eligible interventions during the first thousand days to prevent childhood obesity published between 2016 and 2021. [file 12889_2022_14701_MOESM1_ESM.docx]

|  | Terms | MeSH |
| --- | --- | --- |
| Situation | Obesity | Overweight  Body mass index  Body weight  Obes*  Body fat  Adiposity  Body mass |
| Population | First 1000 days | First 1000 days  First thousand days  Pregnan*  Neonate*  Epigenetic Programm*  Early Programm*  Fetal Programm*  Baby  Babies  Infant  Newborn* |
| Action | Intervention | Intervention  Prevention  Clinical trial  Promotion  Primary care  Counselling  Education  Program |

**Additional file 1.** Search strategy for databases to identify eligible interventions during the first thousand days to prevent childhood obesity published between 2016 and 2021.

(Overweight OR “Body mass index” OR “Body Weight” OR Obes* OR “Body fat” Or Adiposity OR “Body mass”)

AND

(“First thousand days” OR “First 1000 days” OR Pregnan* OR Neonate* OR “Epigenetic Programm*” OR “Early Programm*” OR “Fetal Programm*” OR Baby OR Babies OR Infant OR Newborn)

AND

(Intervention OR Prevention OR “Clinical trial” OR Promotion OR “Primary care” OR Counselling OR Education OR Program)
