# Supplementary material for: Interventions in the first 1000 days to prevent childhood obesity: a systematic review and quantitative content analysis
Source: BMC Public Health. 2022 Dec 16;22:2367. doi: 10.1186/s12889-022-14701-9 (PMC9758903; doi:10.1186/s12889-022-14701-9)
Supplement: Supplementary file 2 — Additional file 2. List of eligible articles published between 2016 and 2021 detailing an intervention during the first thousand days to prevent childhood obesity. [file 12889_2022_14701_MOESM2_ESM.docx]

**Additional file 2.** List of eligible articles published between 2016 and 2021 detailing an intervention during the first thousand days to prevent childhood obesity.

1. Savage JS, Hohman EE, McNitt KM, Pauley AM, Leonard KS, Turner T, et al. Uncontrolled Eating during Pregnancy Predicts Fetal Growth: The Healthy Mom Zone Trial. Nutrients. 2019;11(4):1–16.
2. Garmendia ML, Casanello P, Flores M, Kusanovic JP, Uauy R. The effects of a combined intervention (docosahexaenoic acid supplementation and home-based dietary counseling) on metabolic control in obese and overweight pregnant women: the MIGHT study. Am J Obstet Gynecol [Internet]. 2021;224(5):526.e1-526.e25. Available from: https://doi.org/10.1016/j.ajog.2020.10.048
3. Liu YQ, Liu Y, Hua Y, Chen XL. Effect of diet and exercise intervention in Chinese pregnant women on gestational weight gain and perinatal outcomes: A quasi-experimental study. Appl Nurs Res [Internet]. 2017;36:50–6. Available from: http://dx.doi.org/10.1016/j.apnr.2017.05.001
4. Horan MK, McGowan CA, Gibney ER, Byrne J, Donnelly JM, McAuliffe FM. Maternal nutrition and glycaemic index during pregnancy impacts on offspring adiposity at 6 months of age—analysis from the ROLO randomised controlled trial. Nutrients. 2016;8(1):13–5.
5. Dodd JM, Louise J, Deussen AR, Grivell RM, Dekker G, McPhee AJ, et al. Effect of metformin in addition to dietary and lifestyle advice for pregnant women who are overweight or obese: the GRoW randomised, double-blind, placebo-controlled trial. Lancet Diabetes Endocrinol [Internet]. 2019;7(1):15–24. Available from: http://dx.doi.org/10.1016/S2213-8587(18)30310-3
6. Garmendia ML, Corvalan C, Araya M, Casanello P, Kusanovic JP, Uauy R. Effectiveness of a normative nutrition intervention in Chilean pregnant women on maternal and neonatal outcomes: The CHiMINCs study. Am J Clin Nutr. 2020;112(4):991–1001.
7. Robertson N, Ladlow B. Effect of individual dietetic intervention on gestational weight gain and associated complications in obese pregnant women. Aust New Zeal J Obstet Gynaecol. 2018;58(3):274–7.
8. Reifsnider E, McCormick DP, Cullen KW, Todd M, Moramarco MW, Gallagher MR, et al. Randomized Controlled Trial to Prevent Infant Overweight in a High-Risk Population. Acad Pediatr [Internet]. 2018 Apr [cited 2019 Apr 1];18(3):324–33. Available from: http://www.ncbi.nlm.nih.gov/pubmed/29277462
9. Parat S, Nègre V, Baptiste A, Valensi P, Bertrand A-M, Chollet C, et al. Prenatal education of overweight or obese pregnant women to prevent childhood overweight (the ETOIG study): an open-label, randomized controlled trial. Int J Obes [Internet]. 2019 Feb 21 [cited 2019 Mar 4];43(2):362–73. Available from: http://www.nature.com/articles/s41366-018-0205-z
10. Gross RS, Mendelsohn AL, Gross MB, Scheinmann R, Messito MJ. Randomized Controlled Trial of a Primary Care-Based Child Obesity Prevention Intervention on Infant Feeding Practices. J Pediatr [Internet]. 2016;174:171-177.e2. Available from: http://dx.doi.org/10.1016/j.jpeds.2016.03.060
11. Gregory EF, Goldshore MA, Henderson JL, Weatherford RD, Showell NN. Infant Growth following Maternal Participation in a Gestational Weight Management Intervention. Child Obes. 2016;12(3):219–25.
12. Kunath J, Günther J, Rauh K, Hoffmann J, Stecher L, Rosenfeld E, et al. Effects of a lifestyle intervention during pregnancy to prevent excessive gestational weight gain in routine care - the cluster-randomised GeliS trial. BMC Med. 2019;17(1):1–13.
13. Okesene-Gafa KAM, Li M, McKinlay CJD, Taylor RS, Rush EC, Wall CR, et al. Effect of antenatal dietary interventions in maternal obesity on pregnancy weight-gain and birthweight: Healthy Mums and Babies (HUMBA) randomized trial. Am J Obstet Gynecol. 2019 Aug 1;221(2):152.e1-152.e13.
14. Huang RC, Silva D, Beilin L, Neppe C, Mackie KE, Roffey E, et al. Feasibility of conducting an early pregnancy diet and lifestyle e-health intervention: The Pregnancy Lifestyle Activity Nutrition (PLAN) project. J Dev Orig Health Dis. 2020;11(1):58–70.
15. Rönö K, Grotenfelt NE, Klemetti MM, Stach-Lempinen B, Huvinen E, Meinilä J, et al. Effect of a lifestyle intervention during pregnancy—findings from the Finnish gestational diabetes prevention trial (RADIEL). J Perinatol [Internet]. 2018;38(9):1157–64. Available from: http://dx.doi.org/10.1038/s41372-018-0178-8
16. McDonald SM, Isler C, Haven K, Newton E, Kuehn D, Kelley G, et al. Moderate intensity aerobic exercise during pregnancy and 1-month infant Morphometry. Birth Defects Res. 2021;113(3):238–47.
17. Brownfoot FC, Davey MA, Kornman L. Routine weighing to reduce excessive antenatal weight gain: A randomised controlled trial. BJOG An Int J Obstet Gynaecol. 2016;123(2):254–61.
18. Ortiz-Félix RE, Cárdenas-Villarreal VM, Miranda-Félix PE, Guevara-Valtier MC. Impacto de una intervención de educación prenatal en mujeres embarazadas para prevenir el sobrepeso en lactantes. Gac M�xico. 2021;157(1):3–9.
19. Trak-Fellermeier MA, Campos M, Meléndez M, Pomeroy J, Palacios C, Rivera-Viñas J, et al. Pearls randomized lifestyle trial in pregnant hispanic women with overweight/obesity: Gestational weight gain and offspring birthweight. Diabetes, Metab Syndr Obes Targets Ther. 2019;12:225–38.
20. Whyte K, Johnson J, Kelly K, Horowitz M, Widen EM, Toro-Ramos T, et al. No sustained effects of an intervention to prevent excessive GWG on offspring fat and lean mass at 54 weeks: Yet a greater head circumference persists. Pediatr Obes. 2021;16(7):1–8.
21. Haby K, Berg M, Gyllensten H, Hanas R, Premberg Å. Mighty Mums - a lifestyle intervention at primary care level reduces gestational weight gain in women with obesity. BMC Obes. 2018;5(1):1–12.
22. Phelan S, Hart CN, Jelalian E, Muñoz-Christian K, Alarcon N, McHugh A, et al. Effect of prenatal lifestyle intervention on maternal postpartum weight retention and child body mass index z-score at 36 months. Int J Obes [Internet]. 2021;45(5):1133–42. Available from: http://dx.doi.org/10.1038/s41366-021-00784-8
23. McDonald SM, Yeo SA, Liu J, Wilcox S, Sui X, Pate RR. Associations between maternal physical activity and fitness during pregnancy and infant birthweight. Prev Med Reports [Internet]. 2018;11(December 2017):1–6. Available from: https://doi.org/10.1016/j.pmedr.2018.04.019
24. Dodd JM, Deussen AR, Mohamad I, Rifas-Shiman SL, Yelland LN, Louise J, et al. The effect of antenatal lifestyle advice for women who are overweight or obese on secondary measures of neonatal body composition: The LIMIT randomised trial. BJOG An Int J Obstet Gynaecol. 2016;123(2):244–53.
25. Liu J, Wilcox S, Wingard E, Turner-McGrievy G, Hutto B, Burgis J. A Behavioral Lifestyle Intervention to Limit Gestational Weight Gain in Pregnant Women with Overweight and Obesity. Obesity. 2021;29(4):672–80.
26. Taylor RW, Gray AR, Heath ALM, Galland BC, Lawrence J, Sayers R, et al. Sleep, nutrition, and physical activity interventions to prevent obesity in infancy: Follow-up of the Prevention of Overweight in Infancy (POI) randomized controlled trial at ages 3.5 and 5 y. Am J Clin Nutr. 2018;108(2):228–36.
27. Wang C, Wei Y, Zhang X, Zhang Y, Xu Q, Sun Y, et al. A randomized clinical trial of exercise during pregnancy to prevent gestational diabetes mellitus and improve pregnancy outcome in overweight and obese pregnant women. Am J Obstet Gynecol. 2017;216(4):340–51.
28. Dorise B, Byth K, McGee T, Wood A, Blumenthal C. A low intensity dietary intervention for reducing excessive gestational weight gain in an overweight and obese pregnant cohort. Eat Weight Disord [Internet]. 2020;25(2):257–63. Available from: http://dx.doi.org/10.1007/s40519-018-0566-2
29. Peccei A, Blake-Lamb T, Rahilly D, Hatoum I, Bryant A. Intensive Prenatal Nutrition Counseling in a Community Health Setting. Obstet Gynecol [Internet]. 2017 Aug [cited 2019 Apr 3];130(2):423–32. Available from: http://www.ncbi.nlm.nih.gov/pubmed/28697099
30. Seneviratne SN, Jiang Y, Derraik JGB, McCowan LME, Parry GK, Biggs JB, et al. Effects of antenatal exercise in overweight and obese pregnant women on maternal and perinatal outcomes: A randomised controlled trial. BJOG An Int J Obstet Gynaecol. 2016;123(4):588–97.
31. Lindberg SM, DeBoth A, Anderson CK. Effect of a Best Practice Alert on Gestational Weight Gain, Health Services, and Pregnancy Outcomes. Matern Child Health J. 2016;20(10):2169–78.
32. Sagedal LR, Øverby NC, Bere E, Torstveit MK, Lohne-Seiler H, Småstuen M, et al. Lifestyle intervention to limit gestational weight gain: the Norwegian Fit for Delivery randomised controlled trial. BJOG An Int J Obstet Gynaecol. 2017;124(1):97–109.
33. Ronnberg A-K, Hanson U, Nilsson K. Effects of an antenatal lifestyle intervention on offspring obesity - a 5-year follow-up of a randomized controlled trial. Acta Obstet Gynecol Scand [Internet]. 2017 Sep 1 [cited 2019 Apr 3];96(9):1093–9. Available from: http://doi.wiley.com/10.1111/aogs.13168
34. Chwah SR, Reilly A, Hall B, O’Sullivan AJ, Henry A. Engagement with and outcomes of a Midwifery-led intervention group for pregnant women of high body mass index. Obstet Med. 2016;9(3):120–5.
35. Dodd JM, Deussen AR, Louise J. A randomised trial to optimise gestational weight gain and improve maternal and infant health outcomes through antenatal dietary, lifestyle and exercise advice: The OPTIMISE randomised trial. Nutrients. 2019;11(12).
36. Soliman AZM, Hassan A, Fahmy HH, Abdelsalam AE, Salem MAA. Maternal and fetal outcomes of pregnant females after a nutritional health education program. An interventional study. Prog Nutr. 2019;21(4):1063–70.
37. Dalrymple K V., Tydeman FAS, Taylor PD, Flynn AC, O’Keeffe M, Briley AL, et al. Adiposity and cardiovascular outcomes in three-year-old children of participants in UPBEAT, an RCT of a complex intervention in pregnant women with obesity. Pediatr Obes. 2021;16(3):1–11.
38. Li J, Liu J, Zhang C, Liu G, Leng J, Wang L, et al. Effects of lifestyle intervention of maternal gestational diabetes mellitus on offspring growth pattern before two years of age. Diabetes Care. 2021;44(3):e42–4.
39. Figueroa EM, Nitti K, Sladek SM. Lowering gestational diabetes risk by prenatal weight gain counseling. J Am Board Fam Med. 2020;33(2):189–97.
40. Wang L, Mei Z, Li H, Zhang Y, Liu J, Serdula MK. Modifying effects of maternal Hb concentration on infant birth weight in women receiving prenatal iron-containing supplements: A randomised controlled trial. Br J Nutr. 2016;115(4):644–9.
41. Thomson JL, Tussing-Humphreys LM, Goodman MH, Olender SE. Gestational Weight Gain: Results from the Delta Healthy Sprouts Comparative Impact Trial. J Pregnancy. 2016;2016.
42. Pawalia A, Kulandaivelan S, Savant S, Singh-Yadav V. Effect of behavioural interventions for obesity prevention in pregnancy on the adequacy of gestational weight gain and retention: metabolic health of indian women. Serbian Journal of Experimental and Clinical Research [Internet]. 2018 [cited 2022 Mar 16]; Available from: https://sciendo.com/pdf/10.2478/sjecr-2018-0068
43. Bacchi, M., Mottola, M. F., Perales, M., Refoyo, I., & Barakat, R. (2018). Aquatic Activities During Pregnancy Prevent Excessive Maternal Weight Gain and Preserve Birth Weight: A Randomized Clinical Trial. American Journal of Health Promotion : AJHP, 32(3), 729–735. https://doi.org/10.1177/0890117117697520
44. Gallagher, D., Rosenn, B., Toro-Ramos, T., Paley, C., Gidwani, S., Horowitz, M., … Pi-Sunyer, X. (2018). Greater Neonatal Fat-Free Mass and Similar Fat Mass Following a Randomized Trial to Control Excess Gestational Weight Gain. Obesity (Silver Spring, Md.), 26(3), 578–587. https://doi.org/10.1002/OBY.22079
45. Helle, C., Hillesund, E. R., Wills, A. K., & Øverby, N. C. (2019). Evaluation of an eHealth intervention aiming to promote healthy food habits from infancy -the Norwegian randomized controlled trial Early Food for Future Health. The International Journal of Behavioral Nutrition and Physical Activity, 16(1). https://doi.org/10.1186/S12966-018-0763-4
46. Klein, J. D., Gorzkowski, J., Resnick, E. A., Harris, D., Kaseeska, K., Pbert, L., … Wasserman, R. (2020). Prenatal and Pediatric Primary Care-Based Child Obesity Prevention Program: A Randomized Trial. Pediatrics, 146(4). https://doi.org/10.1542/PEDS.2020-0709
47. Kouwenhoven, S. M. P., Antl, N., Finken, M. J. J., Twisk, J. W. R., van der Beek, E. M., Abrahamse-Berkeveld, M., … Koletzko, B. V. (2021). Long-term effects of a modified, low-protein infant formula on growth and body composition: Follow-up of a randomized, double-blind, equivalence trial. Clinical Nutrition, 40(6), 3914–3921. https://doi.org/10.1016/J.CLNU.2021.04.034
48. Lovell, A. L., Milne, T., Matsuyama, M., Hill, R. J., Davies, P. S. W., Grant, C. C., & Wall, C. R. (2021). Protein Intake, IGF-1 Concentrations, and Growth in the Second Year of Life in Children Receiving Growing Up Milk - Lite (GUMLi) or Cow’s Milk (CM) Intervention. Frontiers in Nutrition, 8. https://doi.org/10.3389/FNUT.2021.666228
49. McEachan, R. R. C., Santorelli, G., Bryant, M., Sahota, P., Farrar, D., Small, N., … Wright, J. (2016). The HAPPY (Healthy and Active Parenting Programmme for early Years) feasibility randomised control trial: Acceptability and feasibility of an intervention to reduce infant obesity. BMC Public Health, 16(1), 1–15. https://doi.org/10.1186/S12889-016-2861-Z/TABLES/3
50. Savage, J. S., Birch, L. L., Marini, M., Anzman-Frasca, S., & Paul, I. M. (2016). Effect of the INSIGHT Responsive Parenting Intervention on Rapid Infant Weight Gain and Overweight Status at Age 1 Year: A Randomized Clinical Trial. JAMA Pediatrics, 170(8), 742–749. https://doi.org/10.1001/JAMAPEDIATRICS.2016.0445
51. Simione, M., Moreno-Galarraga, L., Perkins, M., Price, S. N., Luo, M., Kotelchuck, M., … Taveras, E. M. (2021). Effects of the First 1000 Days Program, a systems-change intervention, on obesity risk factors during pregnancy. BMC Pregnancy and Childbirth, 21(1), 1–9. https://doi.org/10.1186/S12884-021-04210-9/TABLES/2
52. Vlasblom, E., van Grieken, A., Beltman, M., L’Hoir, M. P., Raat, H., & Boere-Boonekamp, M. M. (2020). Parenting support to prevent overweight during regular well-child visits in 0-3 year old children (BBOFT+ program), a cluster randomized trial on the effectiveness on child BMI and health behaviors and parenting. PloS One, 15(8). https://doi.org/10.1371/JOURNAL.PONE.0237564
